# Data on a new biomarker for kidney transplant recipients: The number of FoxP3 regulatory T cells in the circulation

**DOI:** 10.1016/j.dib.2018.11.083

**Published:** 2018-11-27

**Authors:** Francisco Herrera-Gómez, Waldo del Aguila, Armando Tejero-Pedregosa, Marcel Adler, Rosario Padilla-Berdugo, Álvaro Maurtua-Briseño-Meiggs, Julio Pascual, Manuel Pascual, David San Segundo, Sebastiaan Heidt, F. Javier Álvarez, Carlos Ochoa-Sangrador, Claude Lambert

**Affiliations:** a Pharmacology and Therapeutics, Faculty of Medicine, University of Valladolid, Valladolid, Spain; b Nephrology, Hospital Virgen de la Concha – Sanidad de Castilla y León, Zamora, Spain; c Internal Medicine, Kliniken Nordoberpfalz AG, Bayern, Germany; d Intensive Care Medicine, Hospital Virgen de la Concha – Sanidad de Castilla y León, Zamora, Spain; e Hematology Service and Main Hematology Laboratory, Centre Hospitalier Universitaire Vaudois, Lausanne, Switzerland; f Woodland Medical Practice – NHS, Lincolnshire, United Kingdom; g Nephrology, Hospital del Mar, Barcelona, Spain; h Centre de Transplantation d׳Organes, Centre Hospitalier Universitaire Vaudois, Lausanne, Switzerland; i Immunology, Hospital Universitario Marqués de Valdecilla, Santander, Spain; j Immunohaematology and Blood Transfusion, Leiden University Medical Center, Leiden, the Netherlands; k CEIm Área de Salud Valladolid Este, Hospital Clínico Universitario de Valladolid, Valladolid, Spain; l Clinical Epidemiology Research Support, Sanidad de Castilla y León, Zamora, Spain; m Immunology, Centre Hospitalier Universitaire de Saint-Etienne, Saint-Priest-en-Jarez, France

## Abstract

This article presents unrevealed details of the systematic review process of the article “The number of FoxP3 regulatory T cells in the circulation may be a predictive biomarker for kidney transplant recipients: A multistage systematic review” (Herrera-Gómez et al., 2018). Eligibility criteria guiding searches and study selection, the risk of bias assessment, the assessment of medicine-test codependency (evaluation of the body of evidence), and meta-analytic calculations are provided. The data allows other researchers, particularly those involved in experiments on Translational Epidemiology applied to Pharmacology, to corroborate and extend our assessments.

**Specifications table**

**Table:** 

**Subject area**	Biology
**More specific subject area**	Translational pharmacology
**Type of data**	Text, tables, and figures.
**How data was acquired**	Definition of eligibility criteria and search strategy for study selection, risk of bias assessment, assessment of codependent health technologies, and meta-analytic assessment.
**Data Format**	Raw and analyzed.
**Experimental factors**	Systematic review protocol registration, study selection process (against eligibility criteria), and data extraction.
**Experimental features**	Inclusion and exclusion criteria, full search strategy, risk of bias assessment, assessment of medicine–test codependency, and continuous data meta-analysis.
**Data source location**	Valladolid, Spain, 41.654444°, −4.7175°
**Data accessibility**	Data is with this article.
**Related research article**	F. Herrera-Gómez, W. del Aguila, A. Tejero-Pedregosa, M. Adler, R. Padilla-Berdugo, A. Maurtua-Briseño-Meiggs, Julio Pascual, Manuel Pascual, David San Segundo, Sebastiaan Heidt, Javier Álvarez, Carlos Ochoa-Sangrador, Claude Lambert, The Number of FoxP3 Regulatory T Cells in The Circulation May Be a Predictive Biomarker for Kidney Transplant Recipients: A Multistage Systematic Review, Int. Immunopharmacol. 65 (2018) 483–492 [1]

**Value of the data**

• In the field of Translational Pharmacology, sharing systematic review process details is very important.
• This data allows other researchers to corroborate and extend our assessments.
• The main aim of sharing this data is to improve the qualification of potential predictive biomarkers.

## 1 Data

**Fig. 1:**
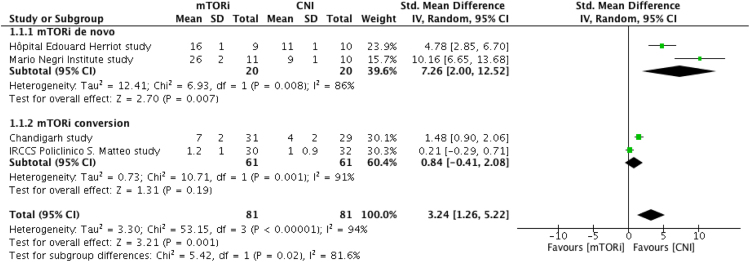
Increase in Tregs at 3–6 months post-transplantation. CI, confidence interval; CNI, calcineurin inhibitor; IV, inverse variance; mTORi, mammalian Target of Rapamycin inhibitor; ST, standard deviation; Tregs, FoxP3 regulatory T cell.

**Fig. 2:**
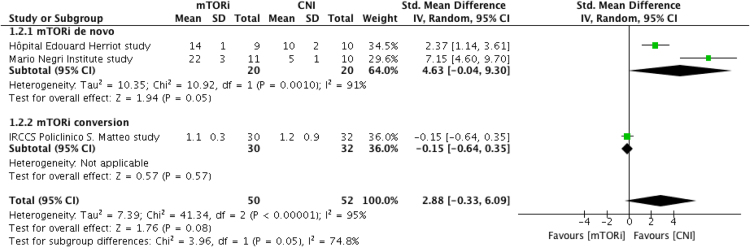
Increase in Tregs at 12 months post-transplantation. CI, confidence interval; CNI, calcineurin inhibitor; IV, inverse variance; mTORi, mammalian Target of Rapamycin inhibitor; ST, standard deviation; Tregs, FoxP3 regulatory T cell.

**Fig. 3:**
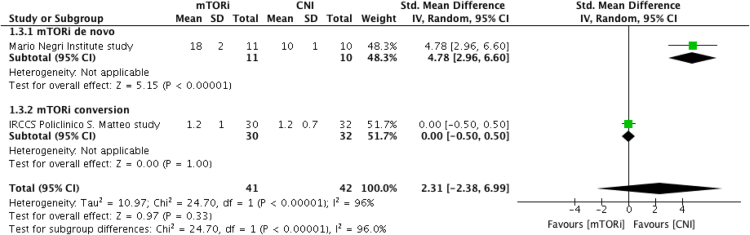
Increase in Tregs at 24 months post-transplantation. CI, confidence interval; CNI, calcineurin inhibitor; IV, inverse variance; mTORi, mammalian Target of Rapamycin inhibitor; ST, standard deviation; Tregs, FoxP3 regulatory T cell.

In addition to links to the four systematic review protocols registered in the International Prospective Register of Systematic Reviews (PROSPERO) (Appendix A. Supplementary material. Text S1), this article presents the inclusion and exclusion criteria ( Tables 1 and 2), the entire search strategy for the systematic reviews performed (Appendix A. Supplementary material. Text S2), the risk of bias (quality) assessment details (Table 3, Table 4, Table 5), the assessment of medicine–test codependency (Table 6), and the meta-analyses (Fig. 1, Fig. 2, Fig. 3) were not included in the article of Herrera-Gómez et al. [1].

**Table 1:** Review questions and study eligibility for each of the 4 systematic reviews.

	**Systematic mapping/systematic review support for**	**In-depth systematic review/systematic review support for**
**Review questions**	aWhat are the changes in the peripheral blood immune phenotype that are associated with COT^b^?	cWhat effect does the increased frequency of regulatory cells in the circulation in KTRs and LTRs have on AR/AAD when using mTORi with/without BELA?
	cWhich tolerance-associated blood cells or regulatory cells increase in the circulation in KTOLs and LTOLs^d^?	eWhat effect does the increase in Tregs in the circulation under mTORi-based IS have on AR/AAD in KTRs?
	fIs there an increased frequency of Tregs in the circulation^g^ in KTOLs?	eWhat is the effect of mTORi-based IS on the number of Tregs in the circulation in KTRs?
		fWhat is the effect on AR/AAD that corresponds to an increased frequency of Tregs in the circulation in KTRs when using mTORi with/without BELA?
**Participants/population**	aPediatric and adult SOTRs.	eAdult KTRs.
	cAdult KTRs or LTRs.	cAdult KTRs or LTRs.
	fAdult KTRs.	fAdult KTRs.
**Intervention(s)/exposures(s)**	aCOT	cThe increase in regulatory cells in the circulation^g^ under mTORi- or mTORi—BELA-based IS.
	cThe increase in regulatory cells in the circulation^g^.	
	fThe increase in Tregs in the circulation^g^.	emTORi-based IS.
		fThe increase in Tregs in the circulation^g^ under mTORi- or mTORi—BELA-based IS.
**Comparators**	aISDs including KTRs with CR.	cDecreased/unchanged numbers of regulatory cells in the circulation^g^ under CNI- or BELA-based IS.
	cDecreased/unchanged numbers of regulatory cells in the circulation^g^.	eCNI-based IS
	fDecreased/unchanged numbers of Tregs in the circulation^g^.	fDecreased/unchanged numbers of Tregs in the circulation^g^ under CNI- or BELA-based IS.
**Outcomes**	aRegulatory cells that increase in KTOLs, LTOLs and other tolerant SOTRs.	c, e, fLess AR/AAD events.
	c, fCOT.	eThe increase in Tregs in the circulation.
**Study design**	Prognostic studies^h^	RCT

Abbreviations: AR/AAD, acute rejection-associated acute allograft dysfunction; BELA, belatacept; CNI, calcineurin inhibitor; COT, clinical operational tolerance; CR, chronic rejection; IS, immunosuppression; ISD, immunosuppression dependent recipient; KTOL, tolerant kidney recipient; KTR, kidney transplant recipient; LTOL, tolerant liver recipient; LTR, liver transplant recipient; mTORi, mammalian Target Of Rapamycin inhibitor; RCT, randomized controlled trial; SOTR, solid organ transplant recipient; Treg, FoxP3 regulatory T cell.

a One-stage systematic review to support the core systematic mapping (CRD42018084941).

b The state in which recipients exhibits a well-functioning graft and lacks histological signs of rejection after being completely off all immunosuppression for at least 1 year.

c Core two-stage systematic review constituted of a systematic mapping followed by an in-depth systematic review (CRD42017057570).

d Increased frequency of Tregs in the circulation are observed in KTOLs and LTOLs, an increase in transitional B cells and other B cells are seen only in KTOLs, and increased γδ T cells are observed only in LTOLs.

e One-stage systematic review to support the core in-depth systematic review (CRD42018085186).

f In-focus two-stage systematic review of the same design as the core two-stage systematic review (CRD4201808085019).

g Increased and decreased numbers of cells for each regulatory cell population were defined by the authors of the included studies according to marker sets for the flow cytometric analysis of these populations.

h Prospective and retrospective comparative cohort studies.

**Table 2:** Exclusion criteria.

**Overall**
• in vivo (animal) and in-vitro studies • Non-systematic and systematic reviews
**Systematic mapping/systematic review support for**
Only involving KTRs:
• No analysis of immune cell phenotypes (flow cytometry) • RCTs
**In-depth systematic review/systematic review support for**
Only involving KTRs:
• No quantification of Tregs (flow cytometry) • No CNI in control groups • No measurement of the outcome of AAD • Comparative and non-comparative cohort (observational) studies

Abbreviations: AAD, acute allograft dysfunction; CNI, calcineurin inhibitor; KTR, kidney transplant recipient; RCT, randomized controlled trial; Treg, FoxP3 regulatory T cell.

**Table 3:** Operationalization of the QUIPS tool bias items for assessing risk of bias in prognostic studies.

**Potential bias**	**Items to be considered for assessment potential opportunities of bias**
**Study participation**The study sample adequately represents the population of interest.	• There is adequate participation in the study by eligible individuals (kidney recipients). • The source population or population of interest is adequately described (demographic and transplantation details). • The sampling frame and recruitment, period of recruitment, and place of recruitment (setting and geographic location) are adequately described. • Inclusion and exclusion criteria are adequately described.
**Study attrition**The study data available (i.e., participants not lost to follow-up) adequately represents the study sample.	• Response rate (i.e., proportion of study sample completing the study and providing outcome data) is adequate. • Attempts to collect information on participants who dropped out of the study are described, and reasons for loss to follow-up are provided. • Participants lost to follow-up are adequately described
**Prognostic factor measurement**	• A clear definition or description of the prognostic factor measured (i.e., the changes in the immune phenotype associated with operational tolerance) is provided. • Continuous variables are reported and appropriate (i.e., not data-dependent) cut-points are used. • The prognostic factor measurement and methods are adequately valid and reliable. • An adequate proportion of the study sample has complete data for the prognostic factor. • The method and setting of measurement are the same for all study participants.
The prognostic factor of interest is measured similarly for all participants.	
**Outcome measurement**	• A clear definition of the outcome of interest (i.e., clinical operational tolerance after kidney transplantation) is provided. • The outcome measures and methods used are adequately valid and reliable (and may include characteristics, such as blind measurement and confirmation of outcome with a valid and reliable test). • The method and setting of measurement are the same for all study participants.
The outcome of interest is measured similarly for all participants.	
**Confounding measurement and account**	• All confounders, including treatments, are measured. • Clear definitions of the important confounders measured are provided (e.g., including dose, level, and duration of exposures). • The measurement of all important confounders is adequately valid and reliable. • The method and setting of confounding measurement is the same for all study participants. • Appropriate methods are used if imputation is used for missing confounder data. • Important potential confounders are accounted for in the study design (e.g., matching for key variables, stratification, and initial assembly of comparable groups). • Important potential confounders are accounted for in the analysis (e.g., appropriate adjustment).
Important potential confounding factors are appropriately accounted for	
**Statistical analysis and reporting**	• There is sufficient presentation of data to assess the adequacy of the analysis. • The strategy for model building (i.e., inclusion of variables) is appropriate and is based on a conceptual framework or model. • The selected model is adequate for the design of the study. • There is no selective reporting of results.
The statistical analysis is appropriate, and all primary outcomes are reported

**Table 4:** Assessing risk of bias in eligible prognostic studies eligible using the QUIPS tool.

**Studies**	**Study participation**	**Study attrition**	**Prognostic factor measurement**	**Outcome measurement**	**Confounding measurement and account**	**Statistical analysis and reporting**
**King׳s College London study**^b^	Low risk of bias	Moderate risk of bias	High risk of bias	Low risk of bias	High risk of bias	Low risk of bias
**ITN507 (FACTOR)**^c^	Low risk of bias	Moderate risk of bias	High risk of bias	Low risk of bias	High risk of bias	Low risk of bias
**Nantes study**^d^	Low risk of bias	Moderate risk of bias	Moderate risk of bias	Low risk of bias	High risk of bias	Low risk of bias
**BMOTS**^a^	Low risk of bias	Low risk of bias	Moderate risk of bias	Low risk of bias	High risk of bias	Low risk of bias

Abbreviations: INSERM, Institut National de la Santé Et de la Recherche Médicale; IOT, Indices Of Tolerance; ITN, Immune Tolerance Network.

a BMOTS, the Brazilian Multicenter Operational Tolerance study.

b IOT consortium study.

c ITN study.

d INSERM study.

**Table 5:** Assessing risk of bias in eligible trials eligible using the Cochrane risk of bias tool.

**Trials**	**Random sequence generation**	**Allocation concealment**	**Blinding of participants and personnel**	**Blinding of outcome assessment**	**Incomplete outcome data**	**Selective reporting**	**Other bias**
**Mario Negri Institute study**	Low risk of bias	Low risk of bias	Unclear risk of bias	Unclear risk of bias	Low risk of bias	Unclear risk of bias	Low risk of bias
**Hôpital Edouard Herriot study**	Low risk of bias	Low risk of bias	Unclear risk of bias	Unclear risk of bias	Unclear risk of bias	Unclear risk of bias	Unclear risk of bias
**Chandigarh study**	Low risk of bias	Low risk of bias	Unclear risk of bias	Unclear risk of bias	Low risk of bias	Unclear risk of bias	Unclear risk of bias
**University of Foggia study**	Low risk of bias	Low risk of bias	Unclear risk of bias	Unclear risk of bias	Unclear risk of bias	Unclear risk of bias	Low risk of bias
**IRCCS Policlinico S. Matteo study**^a^	Low risk of bias	Low risk of bias	Unclear risk of bias	Unclear risk of bias	Low risk of bias	Low risk of bias	Low risk of bias
**BMS-224818 study**^b^	Low risk of bias	Low risk of bias	Unclear risk of bias	Unclear risk of bias	Low risk of bias	Low risk of bias	Unclear risk of bias

Abbreviations: IRCCS, Istituto di ricovero e cura a carattere scientifico.

a Fondazione IRCCS Policlinico San Matteo study.

b Bristol-Myers Squibb study.

**Table 6:** Adaptation of the Merlin׳s tool to assess codependency in the combination of treatment and test.

**Information requests**	**Comments**
**Section 1 – Context**	
**Details about the biomarker, the test and the medicine**	
1 (O) Current reimbursement arrangements.	The medicines and the test are affordable in developed countries, and are available in more and more developing countries.
2 (T) Test sponsor.	Becton, Dickinson and Company (BD).
3 (M) Medicine sponsor.	SIR (Pfizer: Rapamune®).
	BELA (Bristol-Myers-Squibb: Nulojix®)
4 (O) Biomarker.	The number of Tregs in the circulation.
5 (T) Proposed test.	Quantification of circulating Tregs by flow cytometry
6 (O) Medical condition or problem being managed.	AR/AADs in KTRs.
7 (O) Clinical management pathways.	Monitoring of patients.
**Rationale for the codependency**	
8 (O) Definition of the biomarker.	Increased/decreased Tregs in the circulation.
9 (O) Biological rationale for targeting specific biomarker(s).	Patients with increased Tregs presented less frequent AR/AADs.
10 (O) Other biomarker(s) to assess treatment effect of the medicine.	None.
11(O) Prevalence of the condition being targeted in the population that is likely to receive the test.	10%
**Proposed impact of codependent technologies on current clinical practice**	
12 (T) Consistency of the test results over time.	Increased Tregs are observed preferentially in KTRs receiving mTORi with/without BELA.
13 (T) Use of the proposed test with other treatments and/or for other purposes.	NA
14 (T) Use of the test in the clinical management pathway.	The test is most likely to be an additional test for managing patients.
15 (T) Provision of the test.	The test is routinely used in hospitals of developed countries.
16 (T) Specimen or sample collection.	Peripheral blood.
17 (T) Use of the test for monitoring purposes (if relevant)	Detection of patients at high risk for AR/AADs.
18(O) Availability of other tests for the biomarker.	None.
**Section 2 – Clinical evaluation**	
**Direct evidence approach**	
**Section 2a Evidence of prognostic effect of the biomarker**	
19(O) Prognostic effect of the biomarker.	It can be assumed methodologically.
**Section 2d Clinical evaluation of the codependent technologies (combined)**	
20(O) Selection of the direct evidence.	Low-level direct evidence is available (retrospective biomarker-stratified trials).
21(O) Quality of the direct evidence.	The evidence is of adequate quality.

Item numbers are tagged with (T), (M) or (O), which indicate whether the item number is relevant to the test, the medicine or overlaps both. Abbreviations: AR/AAD, acute rejection-associated acute allograft dysfunction; BELA, belatacept; mTORi, mammalian Target Of Rapamycin inhibitor; KTR, kidney transplant recipient; SIR, sirolimus.

## 2 Experimental design, materials and methods

For study selection, definition of inclusion and exclusion criteria and the full search strategy were based on the PICOS elements (participants/population, intervention(s)/exposure(s), comparators, outcomes and study design) [1]. The operationalization of the Quality in Prognosis Studies (QUIPS) tool was necessary (Table 3) [2], [3]. Nevertheless, for the risk of bias assessment, the QUIPS tool and the Cochrane Collaboration tool [4] were used when appropriate. For the assessment of medicine–test codependency, an adaptation of Merlin׳s tool included in the guidelines for preparing a submission to the Pharmaceutical Benefits Advisory Committee (PBAC) from the Department of health of Australia was used [5], [6]. Finally, meta-analytic calculations on continuous outcomes (standardized mean-difference effect sizes obtained under inverse variance random-effects model) were performed.
