# Supplementary material for: Data on a new biomarker for kidney transplant recipients: The number of FoxP3 regulatory T cells in the circulation
Source: Data Brief. 2018 Nov 27;21:2567–75. doi: 10.1016/j.dib.2018.11.083 (PMC6288408; doi:10.1016/j.dib.2018.11.083)
Supplement: Supplementary file 1 — Supplementary material [file mmc1.docx]

**Conflict of interest**

The authors declare that they have no conflict of interest to this work.
