# Supplementary material for: Data on a new biomarker for kidney transplant recipients: The number of FoxP3 regulatory T cells in the circulation
Source: Data Brief. 2018 Nov 27;21:2567–75. doi: 10.1016/j.dib.2018.11.083 (PMC6288408; doi:10.1016/j.dib.2018.11.083)
Supplement: Supplementary file 2 — Supplementary material [file mmc2.docx]

Appendix A. Supplementary material for:

**Crucial data for the article of Herrera-Gómez et al.: The number of FoxP3 regulatory T cells in the circulation may be a predictive biomarker for kidney transplant recipients: A multistage systematic review**

Francisco Herrera-Gómez, Waldo del Aguila, Armando Tejero-Pedregosa, Marcel Adler, Rosario Padilla-Berdugo, Álvaro Maurtua-Briseño-Meiggs, Julio Pascual, Manuel Pascual, David San Segundo, Sebastiaan Heidt, F. Javier Álvarez, Carlos Ochoa-Sangrador, Claude Lambert

**Text S1**

**Study notation**

Systematic review registry:

International Prospective Register of Systematic Reviews (PROSPERO)

Registration IDs / links:

CRD42017057570 / <http://www.crd.york.ac.uk/PROSPERO/display_record.php?ID=CRD42017057570>

CRD42018085019 / <http://www.crd.york.ac.uk/PROSPERO/display_record.php?ID=CRD42018085019>

CRD42018084941 / <http://www.crd.york.ac.uk/PROSPERO/display_record.php?ID=CRD42018084941>

CRD42018085186 / <http://www.crd.york.ac.uk/PROSPERO/display_record.php?ID=CRD42018085186>

**Text S2**

**Search strategy**

**Core two-stage systematic review** (PROSPERO registration ID: CRD42017057570)

Systematic mapping

**PubMed**

109407: (((("t lymphocytes, regulatory"[MeSH Terms]) OR "b lymphocytes, regulatory"[MeSH Terms]) OR "intraepithelial lymphocytes"[MeSH Terms]) OR "dendritic cells"[MeSH Terms]) OR "killer cells, natural"[MeSH Terms]

50037: (((("treg"[Text Word]) OR "breg"[Text Word]) OR "gamma delta t cell"[Text Word]) OR "dendritic cell"[Text Word]) OR "nk cell"[Text Word]

2292: "Transplantation Tolerance"[Mesh]

3430: ("immune tolerance"[MeSH Terms]) AND "organ transplantation"[MeSH Terms]

21322: (((("transplantation"[Text Word]) OR "transplant"[Text Word]) OR "operational"[Text Word])) AND "tolerance"[Text Word]

**OvidMedline(R)**

**Journals@Ovid (Sumarios y Resúmenes todas las Revistas Ovid), Revistas Subscritas a Texto Completo por Sacyl, OvidMEDLINE(R) Epub Ahead of Print, In-Process & Other Non-Indexed Citantions, Ovid MEDLINE(R) Daily, Ovid MEDLINE and Versions(R)**

109411: exp dendritic cells/ or exp killer cells, natural/ or exp intraepithelial lymphocytes/ or exp t-lymphocytes, regulatory/ or exp b-lymphocytes, regulatory/

38652: (treg or breg or "gamma adj delta adj t adj cell" or "dendritic adj cell" or "nk adj cell").tw.

2292: exp Transplantation Tolerance/

3432: exp Immune Tolerance/ and exp Organ Transplantation/

136227: ((transpl$ or operat$) and tolerance).tw.

**Elsevier’s Scopus**

28553: (KEY("regulatory t cell") OR KEY("regulatory b cell") OR KEY("gamma delta t cell") OR KEY("dendritic cell") OR KEY("natural killer cell")) AND NOT INDEX(medline)

27863: (TITLE-ABS-KEY(treg) OR TITLE-ABS-KEY(breg) OR TITLE-ABS-KEY("gamma delta t cell") OR TITLE-ABS-KEY("dendritic cell") OR TITLE-ABS-KEY("nk cell")) AND NOT INDEX(medline)

481: KEY("transplantation tolerance") AND NOT INDEX(medline)

188: (KEY("immunological tolerance") AND KEY("organ transplantation")) AND NOT INDEX(medline)

30826: ((TITLE-ABS-KEY(transpl*) OR TITLE-ABS-KEY(operat*))) AND (TITLE-ABS-KEY(tolerance)) AND NOT INDEX(medline)

**Web of Science**

**Web of Science Core Collection, Current Contents Connect, Derwent Innovations Index, KCI-Korean Journal Database, Medline, Russian Science Citation Index, SciELO Citation Index**

266360: TOPIC: (treg) OR TOPIC: (breg) OR TOPIC: (gamma delta t cell) OR TOPIC: (dendritic cell) OR TOPIC: (nk cell)

TOPIC: (transpl*) OR TOPIC: (operat*) (a)

TOPIC: (tolerance) (b)

77647: (a) and (b)

**The Cochrane Central Register of Controlled Trials (CENTRAL)**

1240: MeSH descriptor: [T-Lymphocytes, Regulatory] or MeSH descriptor: [B-Lymphocytes, Regulatory] or MeSH descriptor: [Intraepithelial Lymphocytes] or MeSH descriptor: [Dendritic Cells] or MeSH descriptor: [Killer Cells, Natural] explode all trees in Trials

2679: treg:ti,ab,kw or breg:ti,ab,kw or "gamma delta T cell":ti,ab,kw or "dendritic cell":ti,ab,kw or "NK cell":ti,ab,kw in Trials (Word variations have been searched)

18: MeSH descriptor: [Transplantation Tolerance] explode all trees in Trials

36: MeSH descriptor: [Immune Tolerance] and MeSH descriptor: [Organ Transplantation] explode all trees in Trials

"transplantation":ti,ab,kw or "transplant":ti,ab,kw or "operational":ti,ab,kw in Trials (Word variations have been searched) (c)

"tolerance":ti,ab,kw in Trials (Word variations have been searched) (d)

4902: (c) and (d)

**ClinicalTrials.gov, the EU Clinical Trials Register, the United Kingdoms' ISRCTN registry, and the India's Clinical Trial Registry**

802: treg

Also searched for Regulatory T cell and T-regulatory cell in ClinicalTrials.gov

22: breg

Also searched for Regulatory B Cells in ClinicalTrials.gov

50: "gamma delta t cell"

Also searched for Intraepithelial Lymphocytes and Gd T Cells in ClinicalTrials.gov

886: "dendritic cell"

Also searched for Antigen presenting cell in ClinicalTrials.gov

902: "nk cell"

Also searched for Killer Cells in ClinicalTrials.gov

8: "transplantation tolerance"

15: "transplant tolerance"

20: "operational tolerance"

**DART Europe E-Theses**

341: treg

12: breg

10: "gamma delta t cell"

305: "dendritic cell"

327: "nk cell"

11: "transplantation tolerance"

6: "transplant tolerance"

4: "operational tolerance"

**Open Access Theses and Dissertations**

1146: treg

33: breg

31: "gamma delta t cell"

1071: "dendritic cell"

880: "nk cell"

54: "transplantation tolerance"

31: "transplant tolerance"

17: "operational tolerance"

**Meeting abstracts archives**

**The American Association of Immunologists Annual Meeting 2006 to 2017, the European Congress of Immunology 2006, 2009, 2012 and 2015, the American Transplant Congress 2002-2005, 2007-2013, 2015-2017, the World Transplant Congress 2006 and 2014, the European Society for Organ Transplantation (ESOT) Congress 1997, 1999, 2001, 2005, 2007, 2009, 2011, 2013, 2015 and 2017, the Transplantation Society (TTS) International Congress 1998, 2000, 2002, 2004, 2006, 2008, 2010, 2012, 2014 and 2016**

13: manual searches.

In-depth systematic review

**PubMed**

28759: ("t lymphocytes, regulatory"[MeSH Terms]) OR "b lymphocytes, regulatory"[MeSH Terms]

13731: ("treg"[Text Word]) OR "breg"[Text Word]

74200: "kidney function tests"[MeSH Terms]

40239: "glomerular filtration rate"[MeSH Terms]

122674: "creatinine"[Text Word]

317844: "clinical trials as topic"[MeSH Terms]

125730: "controlled clinical trials as topic"[MeSH Terms]

120798: "randomized controlled trials as topic"[MeSH Terms]

808129: "clinical trial"[Publication Type]

557119: "controlled clinical trial"[Publication Type]

469295: "randomized controlled trial"[Publication Type]

1086594: "trial"[Text Word]

95998: "random allocation"[MeSH Terms]

1188673: ((("randomized"[Text Word]) OR "random"[Text Word]) OR "randomly"[Text Word]) OR "randomization"[Text Word]

147670: "double blind method"[MeSH Terms]

25723: "single blind method"[MeSH Terms]

141595: ((("double blind"[Text Word]) OR "double mask"[Text Word]) OR "single blind"[Text Word]) OR "single mask"[Text Word]

36752: "open label"[Text Word]

34076: "placebos"[MeSH Terms]

197944: "placebo"[Text Word]

**OvidMedline(R)**

**Journals@Ovid (Sumarios y Resúmenes todas las Revistas Ovid), Revistas Subscritas a Texto Completo por Sacyl, OvidMEDLINE(R) Epub Ahead of Print, In-Process & Other Non-Indexed Citantions, Ovid MEDLINE(R) Daily, Ovid MEDLINE and Versions(R)**

28760: exp t-lymphocytes, regulatory/ or exp b-lymphocytes, regulatory/

38652: (treg or breg).tw.

74209: exp Kidney Function Tests/

40243: exp Glomerular Filtration Rate/

338764: creatinine.tw.

512552: clinical trial/

92669: controlled clinical trial/

468992: randomized controlled trial/

512552: clinical trial.pt.

92669: controlled clinical trial.pt.

468992: randomized controlled trial.pt.

1588208: trial.tw

95995: Random Allocation/

2758554: random$.tw.

147676: Double-Blind Method/

25718: single-blind method/

404787: ((doubl$ adj blind$) or (doubl$ adj mask$) or (singl$ adj blind$) or (singl$ adj mask$)).tw.

127751: open label.tw.

34079: Placebos/

567003: placebo$.tw.

**Elsevier’s Scopus**

6883: (KEY("regulatory t lymphocyte") OR KEY("regulatory b lymphocyte")) AND NOT INDEX(medline)

4411: (TITLE-ABS-KEY(treg) OR TITLE-ABS-KEY(breg)) AND NOT INDEX(medline)

21483: KEY("kidney function") AND NOT INDEX(medline)

10449: KEY("glomerulus filtration rate") AND NOT INDEX(medline)

49384: TITLE-ABS-KEY(creatinine) AND NOT INDEX(medline)

28031: KEY("clinical trial (topic)") AND NOT INDEX(medline)

1896: KEY("controlled clinical trial (topic)") AND NOT INDEX(medline)

38285: KEY("randomized controlled trial (topic)") AND NOT INDEX(medline)

658544: TITLE-ABS-KEY(trial) AND NOT INDEX(medline)

14531: KEY(randomization) AND NOT INDEX(medline)

1184031: TITLE-ABS-KEY(random*) AND NOT INDEX(medline)

27421: KEY("double blind procedure") AND NOT INDEX(medline)

4686: KEY("single blind procedure") AND NOT INDEX(medline)

54268: (TITLE-ABS-KEY("double blind*") OR TITLE-ABS-KEY("double mask*") OR TITLE-ABS-KEY("single blind*") OR TITLE-ABS-KEY("single mask*")) AND NOT INDEX(medline)

8875: TITLE-ABS-KEY("open label") AND NOT INDEX(medline)

70892: KEY(placebo) AND NOT INDEX(medline)

99519: TITLE-ABS-KEY(placebo) AND NOT INDEX(medline)

**Web of Science**

**Web of Science Core Collection, Current Contents Connect, Derwent Innovations Index, KCI-Korean Journal Database, Medline, Russian Science Citation Index, SciELO Citation Index**

19274: TOPIC: (treg) OR TOPIC: (breg)

180900: TOPIC: (creatinine)

2042190: TOPIC: (trial)

2373510: TOPIC: (random*)

356248: TOPIC: ("double blind*") OR TOPIC: ("double mask*") OR TOPIC: ("single blind*") OR TOPIC: ("single mask*")

62229: TOPIC: ("open label")

312412: TOPIC: (placebo)

**The Cochrane Central Register of Controlled Trials (CENTRAL)**

265: MeSH descriptor: [T-Lymphocytes, Regulatory] or MeSH descriptor: [B-Lymphocytes, Regulatory] explode all trees in Trials

631: treg:ti,ab,kw or breg:ti,ab,kw in Trials (Word variations have been searched)

3887: MeSH descriptor: [Kidney Function Tests] explode all trees in Trials

2475: MeSH descriptor: [Glomerular Filtration Rate] explode all trees in Trials

17543: "creatinine":ti,ab,kw in Trials (Word variations have been searched)

33230: MeSH descriptor: [Clinical Trials as Topic] this term only in Trials

72: MeSH descriptor: [Controlled Clinical Trials as Topic] this term only in Trials

5937: MeSH descriptor: [Randomized Controlled Trials as Topic] this term only in Trials

319398: "clinical trial":pt in Trials (Word variations have been searched)

90524: "controlled clinical trial":pt in Trials (Word variations have been searched)

458934: "randomized controlled trial":pt in Trials (Word variations have been searched)

621290: trial:ti,ab,kw in Trials (Word variations have been searched)

20617: MeSH descriptor: [Random Allocation] this term only in Trials

736635: randomized:ti,ab,kw or random:ti,ab,kw or randomly:ti,ab,kw or "randomization":ti,ab,kw in Trials (Word variations have been searched)

128258: MeSH descriptor: [Double-Blind Method] this term only in Trials

18437: MeSH descriptor: [Single-Blind Method] this term only in Trials

277843: "double blind":ti,ab,kw or "double masked":ti,ab,kw or "single blind":ti,ab,kw or "single masked":ti,ab,kw in Trials (Word variations have been searched)

40918: "open label":ti,ab,kw in Trials (Word variations have been searched)

23339: MeSH descriptor: [Placebos] this term only in Trials

232499: "placebo":ti,ab,kw in Trials (Word variations have been searched)

**ClinicalTrials.gov, the EU Clinical Trials Register, the United Kingdoms' ISRCTN registry, and the India's Clinical Trial Registry**

802: treg

Also searched for Regulatory T cell and T-regulatory cell in ClinicalTrials.gov

22: breg

Also searched for Regulatory B Cells in ClinicalTrials.gov

5638: creatinine

100427: trial

Also searched for Clinical Trials in ClinicalTrials.gov

152577: randomized

Also searched for Randomization in ClinicalTrials.gov

19357: "double blind" or "double mask"

Also searched for Double blind and Double-blinded in ClinicalTrials.gov

1187: "single blind" or "single mask"

Also searched for Single blind in ClinicalTrials.gov

128750: "open label"

54934: placebo

Also searched for Placebo-controlled in ClinicalTrials.gov

**DART Europe E-Theses**

341: treg

12: breg

428: creatinine

4901: trial

2525: randomized

454: "double blind" or "double mask"

66: "single blind" or "single mask"

84: "open label"

1270: placebo

**Open Access Theses and Dissertations**

1146: treg

33: breg

1788: creatinine

28570: trial

17176: randomized

2307: "double blind" or "double mask"

321: "single blind" or "single mask"

282: "open label"

6112: placebo

**Meeting abstracts archives**

**The American Association of Immunologists Annual Meeting 2006 to 2017, the European Congress of Immunology 2006, 2009, 2012 and 2015, the American Transplant Congress 2002-2005, 2007-2013, 2015-2017, the World Transplant Congress 2006 and 2014, the European Society for Organ Transplantation (ESOT) Congress 1997, 1999, 2001, 2005, 2007, 2009, 2011, 2013, 2015 and 2017, the Transplantation Society (TTS) International Congress 1998, 2000, 2002, 2004, 2006, 2008, 2010, 2012, 2014 and 2016**

11: manual searches.

**In-focus two-stage systematic review** (PROSPERO registration ID: CRD42018085019)

Systematic mapping

**PubMed**

90052: "kidney transplantation"[MeSH Terms]

133516: ((("kidney"[Text Word]) OR "renal"[Text Word])) AND "transplantation"[Text Word]

28459: "t lymphocytes, regulatory"[MeSH Terms]

13418: "treg"[Text Word]

2292: "Transplantation Tolerance"[Mesh]

3430: ("immune tolerance"[MeSH Terms]) AND "organ transplantation"[MeSH Terms]

21322: (((("transplantation"[Text Word]) OR "transplant"[Text Word]) OR "operational"[Text Word])) AND "tolerance"[Text Word]

**OvidMedline(R)**

**Journals@Ovid (Sumarios y Resúmenes todas las Revistas Ovid), Revistas Subscritas a Texto Completo por Sacyl, OvidMEDLINE(R) Epub Ahead of Print, In-Process & Other Non-Indexed Citantions, Ovid MEDLINE(R) Daily, Ovid MEDLINE and Versions(R)**

90018: exp Kidney Transplantation/

252747: ((kidney or renal) and transplantation).tw.

28460: exp T-Lymphocytes, Regulatory/

38067: treg.tw.

2292: exp Transplantation Tolerance/

3432: exp Immune Tolerance/ and exp Organ Transplantation/

136227: ((transpl$ or operat$) and tolerance).tw.

**Elsevier’s Scopus**

18266: KEY("kidney transplantation") AND NOT INDEX(medline)

30357: ((TITLE-ABS-KEY(kidney) OR TITLE-ABS-KEY(renal))) AND (TITLE-ABS-KEY(transplantation)) AND NOT INDEX(medline)

1913: KEY("regulatory t cell") AND NOT INDEX(medline)

4275: TITLE-ABS-KEY(treg) AND NOT INDEX(medline)

329: KEY("transplantation tolerance") AND NOT INDEX(medline)

188: (KEY("immunological tolerance") AND KEY("organ transplantation")) AND NOT INDEX(medline)

30846: ((TITLE-ABS-KEY(transpl*) OR TITLE-ABS-KEY(operat*))) AND (TITLE-ABS-KEY(tolerance)) and not INDEX(medline)

**Web of Science**

**Web of Science Core Collection, Current Contents Connect, Derwent Innovations Index, KCI-Korean Journal Database, Medline, Russian Science Citation Index, SciELO Citation Index**

TOPIC: (kidney) OR TOPIC: (renal) (e)

TOPIC: (transplantation) (f)

191179: (e) and (f)

18984: TOPIC: (treg)

TOPIC: (transpl*) OR TOPIC: (operat*) (g)

TOPIC: (tolerance) (h)

77647: (g) and (h)

**The Cochrane Central Register of Controlled Trials (CENTRAL)**

3377: MeSH descriptor: [Kidney Transplantation] explode all trees in Trials

"kidney":ti,ab,kw or "renal":ti,ab,kw in Trials (Word variations have been searched) (i)

"transplantation":ti,ab,kw in Trials (Word variations have been searched) (j)

10212: (i) and (j)

265: MeSH descriptor: [T-Lymphocytes, Regulatory] explode all trees in Trials

623: treg:ti,ab,kw in Trials (Word variations have been searched)

18: MeSH descriptor: [Transplantation Tolerance] explode all trees in Trials

36: MeSH descriptor: [Immune Tolerance] and MeSH descriptor: [Organ Transplantation] explode all trees in Trials

"transplantation":ti,ab,kw or "transplant":ti,ab,kw or "operational":ti,ab,kw in Trials (Word variations have been searched) (k)

"tolerance":ti,ab,kw in Trials (Word variations have been searched) (l)

4902: (k) and (l)

**ClinicalTrials.gov, the EU Clinical Trials Register, the United Kingdoms' ISRCTN registry, and the India's Clinical Trial Registry**

1821: "kidney transplantation"

Also searched for Kidney Transplants, Renal transplant, and Renal Transplantation in ClinicalTrials.gov

802: treg

Also searched for Regulatory T cell and T-regulatory cell in ClinicalTrials.gov

8: "transplantation tolerance"

15: "transplant tolerance"

20: "operational tolerance"

**DART Europe E-Theses**

215: "kidney transplantation"

176: "renal transplantation"

341: treg

11: "transplantation tolerance"

6: "transplant tolerance"

4: "operational tolerance"

**Open Access Theses and Dissertations**

609: "kidney transplantation"

447: "renal transplantation"

1146: treg

54: "transplantation tolerance"

31: "transplant tolerance"

17: "operational tolerance"

**Meeting abstracts archives**

**The American Association of Immunologists Annual Meeting 2006 to 2017, the European Congress of Immunology 2006, 2009, 2012 and 2015, the American Transplant Congress 2002-2005, 2007-2013, 2015-2017, the World Transplant Congress 2006 and 2014, the European Society for Organ Transplantation (ESOT) Congress 1997, 1999, 2001, 2005, 2007, 2009, 2011, 2013, 2015 and 2017, the Transplantation Society (TTS) International Congress 1998, 2000, 2002, 2004, 2006, 2008, 2010, 2012, 2014 and 2016**

12: manual searches.

In-depth systematic review

**PubMed**

90052: "kidney transplantation"[MeSH Terms]

133516: ((("kidney"[Text Word]) OR "renal"[Text Word])) AND "transplantation"[Text Word]

28459: "t lymphocytes, regulatory"[MeSH Terms]

13418: "treg"[Text Word]

18052: "sirolimus"[MeSH Terms]

3988: "everolimus"[MeSH Terms]

22810: "tacrolimus"[Text Word]

28396: "cyclosporine"[MeSH Terms]

94497: (((("sirolimus"[Text Word]) OR "rapamycin"[Text Word]) OR "everolimus"[Text Word]) OR "tacrolimus"[Text Word]) OR "cyclosporine"[Text Word]

402: "belatacept"[Text Word]

74200: "kidney function tests"[MeSH Terms]

40239: "glomerular filtration rate"[MeSH Terms]

122674: "creatinine"[Text Word]

57034: "graft rejection"[MeSH Terms]

14386: "acute rejection"[Text Word]

317844: "clinical trials as topic"[MeSH Terms]

125730: "controlled clinical trials as topic"[MeSH Terms]

120798: "randomized controlled trials as topic"[MeSH Terms]

808129: "clinical trial"[Publication Type]

557119: "controlled clinical trial"[Publication Type]

469295: "randomized controlled trial"[Publication Type]

1086594: "trial"[Text Word]

95998: "random allocation"[MeSH Terms]

1188673: ((("randomized"[Text Word]) OR "random"[Text Word]) OR "randomly"[Text Word]) OR "randomization"[Text Word]

147670: "double blind method"[MeSH Terms]

25723: "single blind method"[MeSH Terms]

141595: ((("double blind"[Text Word]) OR "double mask"[Text Word]) OR "single blind"[Text Word]) OR "single mask"[Text Word]

36752: "open label"[Text Word]

34076: "placebos"[MeSH Terms]

197944: "placebo"[Text Word]

**OvidMedline(R)**

**Journals@Ovid (Sumarios y Resúmenes todas las Revistas Ovid), Revistas Subscritas a Texto Completo por Sacyl, OvidMEDLINE(R) Epub Ahead of Print, In-Process & Other Non-Indexed Citantions, Ovid MEDLINE(R) Daily, Ovid MEDLINE and Versions(R)**

90018: exp Kidney Transplantation/

252747: ((kidney or renal) and transplantation).tw.

28460: exp T-Lymphocytes, Regulatory/

38067: treg.tw.

18052: exp Sirolimus/

3988: exp Everolimus/

14916: exp Tacrolimus/

28398: exp Cyclosporine/

212585: (sirolimus or rapamycin or everolimus or tacrolimus or cyclosporine).tw.

2127: belatacept.tw.

74209: exp Kidney Function Tests/

40243: exp Glomerular Filtration Rate/

338764: creatinine.tw.

57037: exp Graft Rejection/

76980: (acute adj10 rejection).tw.

512552: clinical trial/

92669: controlled clinical trial/

468992: randomized controlled trial/

512552: clinical trial.pt.

92669: controlled clinical trial.pt.

468992: randomized controlled trial.pt.

1588208: trial.tw

95995: Random Allocation/

2758554: random$.tw.

147676: Double-Blind Method/

25718: single-blind method/

404787: ((doubl$ adj blind$) or (doubl$ adj mask$) or (singl$ adj blind$) or (singl$ adj mask$)).tw.

127751: open label.tw.

34079: Placebos/

567003: placebo$.tw.

**Elsevier’s Scopus**

18266: KEY("kidney transplantation") AND NOT INDEX(medline)

30357: ((TITLE-ABS-KEY(kidney) OR TITLE-ABS-KEY(renal))) AND (TITLE-ABS-KEY(transplantation)) AND NOT INDEX(medline)

1913: KEY("regulatory t cell") AND NOT INDEX(medline)

4275: TITLE-ABS-KEY(treg) AND NOT INDEX(medline)

13484: KEY(rapamycin) AND NOT INDEX(medline)

4291: KEY(everolimus) AND NOT INDEX(medline)

11062: KEY(tacrolimus) AND NOT INDEX(medline)

3382: KEY(cyclosporine) AND NOT INDEX(medline)

12169: KEY("cyclosporin A") AND NOT INDEX(medline)

34778: (TITLE-ABS-KEY(sirolimus) OR TITLE-ABS-KEY(rapamycin) OR TITLE-ABS-KEY(everolimus) OR TITLE-ABS-KEY(tacrolimus) OR TITLE-ABS-KEY(cyclosporine)) AND NOT INDEX(medline)

297: TITLE-ABS-KEY(belatacept) AND NOT INDEX(medline)

21469: KEY("kidney function") AND NOT INDEX(medline)

10443: KEY("glomerulus filtration rate") AND NOT INDEX(medline)

49366: TITLE-ABS-KEY(creatinine) AND NOT INDEX(medline)

13144: KEY("graft rejection") AND NOT INDEX(medline)

5086: TITLE-ABS-KEY(acute W/10 rejection) AND NOT INDEX(medline)

28022: KEY("clinical trial (topic)") AND NOT INDEX(medline)

1895: KEY("controlled clinical trial (topic)") AND NOT INDEX(medline)

36252: KEY("randomized controlled trial (topic)") AND NOT INDEX(medline)

658969: TITLE-ABS-KEY(trial) AND NOT INDEX(medline)

14533: KEY(randomization) AND NOT INDEX(medline)

1184854: TITLE-ABS-KEY(random*) AND NOT INDEX(medline)

27401: KEY("double blind procedure") AND NOT INDEX(medline)

4686: KEY("single blind procedure") AND NOT INDEX(medline)

54300: (TITLE-ABS-KEY("double blind*") OR TITLE-ABS-KEY("double mask*") OR TITLE-ABS-KEY("single blind*") OR TITLE-ABS-KEY("single mask*")) AND NOT INDEX(medline)

8888: TITLE-ABS-KEY("open label") AND NOT INDEX(medline)

70864: KEY(placebo) AND NOT INDEX(medline)

99550: TITLE-ABS-KEY(placebo) AND NOT INDEX(medline)

**Web of Science**

**Web of Science Core Collection, Current Contents Connect, Derwent Innovations Index, KCI-Korean Journal Database, Medline, Russian Science Citation Index, SciELO Citation Index**

TOPIC: (kidney) OR TOPIC: (renal) (m)

TOPIC: (transplantation) (n)

191179: (m) and (n)

18984: TOPIC: (treg)

163089: TOPIC: (sirolimus) OR TOPIC: (rapamycin) OR TOPIC: (everolimus) OR TOPIC: (tacrolimus) OR TOPIC: (cyclosporine)

970: TOPIC: (belatacept)

180900: TOPIC: (creatinine)

31310: TOPIC: (acute near/10 rejection)

2042190: TOPIC: (trial)

2373510: TOPIC: (random*)

356248: TOPIC: ("double blind*") OR TOPIC: ("double mask*") OR TOPIC: ("single blind*") OR TOPIC: ("single mask*")

62229: TOPIC: ("open label")

312412: TOPIC: (placebo)

**The Cochrane Central Register of Controlled Trials (CENTRAL)**

3377: MeSH descriptor: [Kidney Transplantation] explode all trees in Trials

"kidney":ti,ab,kw or "renal":ti,ab,kw in Trials (Word variations have been searched) (o)

"transplantation":ti,ab,kw in Trials (Word variations have been searched) (p)

10212: (o) and (p)

265: MeSH descriptor: [T-Lymphocytes, Regulatory] explode all trees in Trials

623: treg:ti,ab,kw in Trials (Word variations have been searched)

1982: MeSH descriptor: [Sirolimus] explode all trees in Trials

1103: MeSH descriptor: [Everolimus] explode all trees in Trials

1750: MeSH descriptor: [Tacrolimus] explode all trees in Trials

730: MeSH descriptor: [Cyclosporine] explode all trees in Trials

12860: "sirolimus":ti,ab,kw or "rapamycin":ti,ab,kw or "everolimus":ti,ab,kw or "tacrolimus":ti,ab,kw or cyclosporine:ti,ab,kw in Trials (Word variations have been searched)

172: belatacept:ti,ab,kw in Trials (Word variations have been searched)

3887: MeSH descriptor: [Kidney Function Tests] explode all trees in Trials

2475: MeSH descriptor: [Glomerular Filtration Rate] explode all trees in Trials

17543: "creatinine":ti,ab,kw in Trials (Word variations have been searched)

2136: MeSH descriptor: [Graft Rejection] explode all trees in Trials

2187: "acute rejection":ti,ab,kw in Trials (Word variations have been searched)

33230: MeSH descriptor: [Clinical Trials as Topic] this term only in Trials

72: MeSH descriptor: [Controlled Clinical Trials as Topic] this term only in Trials

5937: MeSH descriptor: [Randomized Controlled Trials as Topic] this term only in Trials

319398: "clinical trial":pt in Trials (Word variations have been searched)

90524: "controlled clinical trial":pt in Trials (Word variations have been searched)

458934: "randomized controlled trial":pt in Trials (Word variations have been searched)

621290: trial:ti,ab,kw in Trials (Word variations have been searched)

20617: MeSH descriptor: [Random Allocation] this term only in Trials

736635: randomized:ti,ab,kw or random:ti,ab,kw or randomly:ti,ab,kw or "randomization":ti,ab,kw in Trials (Word variations have been searched)

128258: MeSH descriptor: [Double-Blind Method] this term only in Trials

18437: MeSH descriptor: [Single-Blind Method] this term only in Trials

277843: "double blind":ti,ab,kw or "double masked":ti,ab,kw or "single blind":ti,ab,kw or "single masked":ti,ab,kw in Trials (Word variations have been searched)

40918: "open label":ti,ab,kw in Trials (Word variations have been searched)

23339: MeSH descriptor: [Placebos] this term only in Trials

232499: "placebo":ti,ab,kw in Trials (Word variations have been searched)

**ClinicalTrials.gov, the EU Clinical Trials Register, the United Kingdoms' ISRCTN registry, and the India's Clinical Trial Registry**

1821: "kidney transplantation"

Also searched for Kidney Transplants, Renal transplant, and Renal Transplantation in ClinicalTrials.gov

802: treg

Also searched for Regulatory T cell and T-regulatory cell in ClinicalTrials.gov

1638: sirolimus or rapamycin

Also searched for Everolimus, Sirolimus, RAD 001, Temsirolimus, mTOR inhibitor, cci779, Rapamune, Afinitor, Torisen, Certican, Zortess in ClinicalTrials.gov

1342: tacrolimus

Also searched for Prograf and FK 506 in ClinicalTrials.gov

1160: cyclosporine

Also searched for Restasis and Neoral in ClinicalTrials.gov

56: belatacept

Also searched for Nulojix and Bms-224818 in ClinicalTrials.gov

5638: creatinine

694: "acute rejection"

100427: trial

Also searched for Clinical Trials in ClinicalTrials.gov

152577: randomized

Also searched for Randomization in ClinicalTrials.gov

19357: "double blind" or "double mask"

Also searched for Double blind and Double-blinded in ClinicalTrials.gov

1187: "single blind" or "single mask"

Also searched for Single blind in ClinicalTrials.gov

128750: "open label"

54934: placebo

Also searched for Placebo-controlled in ClinicalTrials.gov

**DART Europe E-Theses**

215: "kidney transplantation"

176: "renal transplantation"

341: treg

80: sirolimus or rampamycin

76: everolimus

171: tacrolimus

156: cyclosporine

5: belatacept

428: creatinine

99: "acute rejection"

4901: trial

2525: randomized

454: "double blind" or "double mask"

66: "single blind" or "single mask"

84: "open label"

1270: placebo

**Open Access Theses and Dissertations**

609: "kidney transplantation"

447: "renal transplantation"

1146: treg

1399: sirolimus or rapamycin

140: everolimus

410: tacrolimus

508: cyclosporine

14: belatacept

1788: creatinine

242: "acute rejection"

28570: trial

17176: randomized

2307: "double blind" or "double mask"

321: "single blind" or "single mask"

282: "open label"

6112: placebo

**Meeting abstracts archives**

**The American Association of Immunologists Annual Meeting 2006 to 2017, the European Congress of Immunology 2006, 2009, 2012 and 2015, the American Transplant Congress 2002-2005, 2007-2013, 2015-2017, the World Transplant Congress 2006 and 2014, the European Society for Organ Transplantation (ESOT) Congress 1997, 1999, 2001, 2005, 2007, 2009, 2011, 2013, 2015 and 2017, the Transplantation Society (TTS) International Congress 1998, 2000, 2002, 2004, 2006, 2008, 2010, 2012, 2014 and 2016**

11: manual searches.

**Systematic review support for the core systematic mapping** (PROSPERO registration ID: CRD42018084941)

**PubMed**

2292: "Transplantation Tolerance"[Mesh]

3430: ("immune tolerance"[MeSH Terms]) AND "organ transplantation"[MeSH Terms]

21322: (((("transplantation"[Text Word]) OR "transplant"[Text Word]) OR "operational"[Text Word])) AND "tolerance"[Text Word]

109407: (((("t lymphocytes, regulatory"[MeSH Terms]) OR "b lymphocytes, regulatory"[MeSH Terms]) OR "intraepithelial lymphocytes"[MeSH Terms]) OR "dendritic cells"[MeSH Terms]) OR "killer cells, natural"[MeSH Terms]

50037: (((("treg"[Text Word]) OR "breg"[Text Word]) OR "gamma delta t cell"[Text Word]) OR "dendritic cell"[Text Word]) OR "nk cell"[Text Word]

**OvidMedline(R)**

**Journals@Ovid (Sumarios y Resúmenes todas las Revistas Ovid), Revistas Subscritas a Texto Completo por Sacyl, OvidMEDLINE(R) Epub Ahead of Print, In-Process & Other Non-Indexed Citantions, Ovid MEDLINE(R) Daily, Ovid MEDLINE and Versions(R)**

2292: exp Transplantation Tolerance/

3432: exp Immune Tolerance/ and exp Organ Transplantation/

136227: ((transpl$ or operat$) and tolerance).tw.

109411: exp dendritic cells/ or exp killer cells, natural/ or exp intraepithelial lymphocytes/ or exp t-lymphocytes, regulatory/ or exp b-lymphocytes, regulatory/

38652: (treg or breg or "gamma adj delta adj t adj cell" or "dendritic adj cell" or "nk adj cell").tw.

**Elsevier’s Scopus**

329: KEY("transplantation tolerance") AND NOT INDEX(medline)

188: (KEY("immunological tolerance") AND KEY("organ transplantation")) AND NOT INDEX(medline)

30846: ((TITLE-ABS-KEY(transpl*) OR TITLE-ABS-KEY(operat*))) AND (TITLE-ABS-KEY(tolerance)) AND NOT INDEX(medline)

23516: (KEY("regulatory t cell") OR KEY("regulatory b cell") OR KEY("gamma delta t cell") OR KEY("dendritic cell") OR KEY("natural killer cell")) AND NOT INDEX(medline)

27888: (TITLE-ABS-KEY(treg) OR TITLE-ABS-KEY(breg) OR TITLE-ABS-KEY("gamma delta t cell") OR TITLE-ABS-KEY("dendritic cell") OR TITLE-ABS-KEY("nk cell")) AND NOT INDEX(medline)

**Web of Science**

**Web of Science Core Collection, Current Contents Connect, Derwent Innovations Index, KCI-Korean Journal Database, Medline, Russian Science Citation Index, SciELO Citation Index**

TOPIC: (transpl*) OR TOPIC: (operat*) (q)

TOPIC: (tolerance) (r)

77647: (q) and (r)

266360: TOPIC: (treg) OR TOPIC: (breg) OR TOPIC: (gamma delta t cell) OR TOPIC: (dendritic cell) OR TOPIC: (nk cell)

**The Cochrane Central Register of Controlled Trials (CENTRAL)**

18: MeSH descriptor: [Transplantation Tolerance] explode all trees in Trials

36: MeSH descriptor: [Immune Tolerance] and MeSH descriptor: [Organ Transplantation] explode all trees in Trials

"transplantation":ti,ab,kw or "transplant":ti,ab,kw or "operational":ti,ab,kw in Trials (Word variations have been searched) (s)

"tolerance":ti,ab,kw in Trials (Word variations have been searched) (t)

4902: (s) and (t)

1240: MeSH descriptor: [T-Lymphocytes, Regulatory] or MeSH descriptor: [B-Lymphocytes, Regulatory] or MeSH descriptor: [Intraepithelial Lymphocytes] or MeSH descriptor: [Dendritic Cells] or MeSH descriptor: [Killer Cells, Natural] explode all trees in Trials

2679: treg:ti,ab,kw or breg:ti,ab,kw or "gamma delta T cell":ti,ab,kw or "dendritic cell":ti,ab,kw or "NK cell":ti,ab,kw in Trials (Word variations have been searched)

**ClinicalTrials.gov, the EU Clinical Trials Register, the United Kingdoms' ISRCTN registry, and the India's Clinical Trial Registry**

11: "transplantation tolerance"

6: "transplant tolerance"

4: "operational tolerance"

802: treg

Also searched for Regulatory T cell and T-regulatory cell in ClinicalTrials.gov

22: breg

Also searched for Regulatory B Cells in ClinicalTrials.gov

50: "gamma delta t cell"

Also searched for Intraepithelial Lymphocytes and Gd T Cells in ClinicalTrials.gov

886: "dendritic cell"

Also searched for Antigen presenting cell in ClinicalTrials.gov

902: "nk cell"

Also searched for Killer Cells in ClinicalTrials.gov

**DART Europe E-Theses**

11: "transplantation tolerance"

6: "transplant tolerance"

4: "operational tolerance"

341: treg

12: breg

10: "gamma delta t cell"

305: "dendritic cell"

327: "nk cell"

**Open Access Theses and Dissertations**

54: "transplantation tolerance"

31: "transplant tolerance"

17: "operational tolerance"

1146: treg

33: breg

31: "gamma delta t cell"

1071: "dendritic cell"

880: "nk cell"

**Meeting abstracts archives**

**The American Association of Immunologists Annual Meeting 2006 to 2017, the European Congress of Immunology 2006, 2009, 2012 and 2015, the American Transplant Congress 2002-2005, 2007-2013, 2015-2017, the World Transplant Congress 2006 and 2014, the European Society for Organ Transplantation (ESOT) Congress 1997, 1999, 2001, 2005, 2007, 2009, 2011, 2013, 2015 and 2017, the Transplantation Society (TTS) International Congress 1998, 2000, 2002, 2004, 2006, 2008, 2010, 2012, 2014 and 2016**

14: manual searches.

**Systematic review support for the core in-depth systematic review** (PROSPERO registration ID: CRD42018085186)

**PubMed**

90052: "kidney transplantation"[MeSH Terms]

133516: ((("kidney"[Text Word]) OR "renal"[Text Word])) AND "transplantation"[Text Word]

28459: "t lymphocytes, regulatory"[MeSH Terms]

13418: "treg"[Text Word]

18052: "sirolimus"[MeSH Terms]

3988: "everolimus"[MeSH Terms]

22810: "tacrolimus"[Text Word]

28396: "cyclosporine"[MeSH Terms]

94497: (((("sirolimus"[Text Word]) OR "rapamycin"[Text Word]) OR "everolimus"[Text Word]) OR "tacrolimus"[Text Word]) OR "cyclosporine"[Text Word]

402: "belatacept"[Text Word]

74200: "kidney function tests"[MeSH Terms]

40239: "glomerular filtration rate"[MeSH Terms]

122674: "creatinine"[Text Word]

57034: "graft rejection"[MeSH Terms]

14386: "acute rejection"[Text Word]

**OvidMedline(R)**

**Journals@Ovid (Sumarios y Resúmenes todas las Revistas Ovid), Revistas Subscritas a Texto Completo por Sacyl, OvidMEDLINE(R) Epub Ahead of Print, In-Process & Other Non-Indexed Citantions, Ovid MEDLINE(R) Daily, Ovid MEDLINE and Versions(R)**

90018: exp Kidney Transplantation/

252747: ((kidney or renal) and transplantation).tw.

28460: exp T-Lymphocytes, Regulatory/

38067: treg.tw.

18052: exp Sirolimus/

3988: exp Everolimus/

14916: exp Tacrolimus/

28398: exp Cyclosporine/

212585: (sirolimus or rapamycin or everolimus or tacrolimus or cyclosporine).tw.

2127: belatacept.tw.

74209: exp Kidney Function Tests/

40243: exp Glomerular Filtration Rate/

338764: creatinine.tw.

57037: exp Graft Rejection/

76980: (acute adj10 rejection).tw.

**Elsevier’s Scopus**

18266: KEY("kidney transplantation") AND NOT INDEX(medline)

30357: ((TITLE-ABS-KEY(kidney) OR TITLE-ABS-KEY(renal))) AND (TITLE-ABS-KEY(transplantation)) AND NOT INDEX(medline)

1913: KEY("regulatory t cell") AND NOT INDEX(medline)

4275: TITLE-ABS-KEY(treg) AND NOT INDEX(medline)

13484: KEY(rapamycin) AND NOT INDEX(medline)

4291: KEY(everolimus) AND NOT INDEX(medline)

11062: KEY(tacrolimus) AND NOT INDEX(medline)

3382: KEY(cyclosporine) AND NOT INDEX(medline)

12169: KEY("cyclosporin A") AND NOT INDEX(medline)

34778: (TITLE-ABS-KEY(sirolimus) OR TITLE-ABS-KEY(rapamycin) OR TITLE-ABS-KEY(everolimus) OR TITLE-ABS-KEY(tacrolimus) OR TITLE-ABS-KEY(cyclosporine)) AND NOT INDEX(medline)

297: TITLE-ABS-KEY(belatacept) AND NOT INDEX(medline)

21469: KEY("kidney function") AND NOT INDEX(medline)

10443: KEY("glomerulus filtration rate") AND NOT INDEX(medline)

49366: TITLE-ABS-KEY(creatinine) AND NOT INDEX(medline)

13144: KEY("graft rejection") AND NOT INDEX(medline)

5086: TITLE-ABS-KEY(acute W/10 rejection) AND NOT INDEX(medline)

**Web of Science**

**Web of Science Core Collection, Current Contents Connect, Derwent Innovations Index, KCI-Korean Journal Database, Medline, Russian Science Citation Index, SciELO Citation Index**

TOPIC: (kidney) OR TOPIC: (renal) (u)

TOPIC: (transplantation) (v)

191179: (u) and (v)

18984: TOPIC: (treg)

163089: TOPIC: (sirolimus) OR TOPIC: (rapamycin) OR TOPIC: (everolimus) OR TOPIC: (tacrolimus) OR TOPIC: (cyclosporine)

970: TOPIC: (belatacept)

180900: TOPIC: (creatinine)

31310: TOPIC: (acute near/10 rejection)

**The Cochrane Central Register of Controlled Trials (CENTRAL)**

3377: MeSH descriptor: [Kidney Transplantation] explode all trees in Trials

"kidney":ti,ab,kw or "renal":ti,ab,kw in Trials (Word variations have been searched) (w)

"transplantation":ti,ab,kw in Trials (Word variations have been searched) (x)

10212: (w) and (x)

265: MeSH descriptor: [T-Lymphocytes, Regulatory] explode all trees in Trials

623: treg:ti,ab,kw in Trials (Word variations have been searched)

1982: MeSH descriptor: [Sirolimus] explode all trees in Trials

1103: MeSH descriptor: [Everolimus] explode all trees in Trials

1750: MeSH descriptor: [Tacrolimus] explode all trees in Trials

730: MeSH descriptor: [Cyclosporine] explode all trees in Trials

12860: "sirolimus":ti,ab,kw or "rapamycin":ti,ab,kw or "everolimus":ti,ab,kw or "tacrolimus":ti,ab,kw or cyclosporine:ti,ab,kw in Trials (Word variations have been searched)

172: belatacept:ti,ab,kw in Trials (Word variations have been searched)

3887: MeSH descriptor: [Kidney Function Tests] explode all trees in Trials

2475: MeSH descriptor: [Glomerular Filtration Rate] explode all trees in Trials

17543: "creatinine":ti,ab,kw in Trials (Word variations have been searched)

2136: MeSH descriptor: [Graft Rejection] explode all trees in Trials

2187: "acute rejection":ti,ab,kw in Trials (Word variations have been searched)

**ClinicalTrials.gov, the EU Clinical Trials Register, the United Kingdoms' ISRCTN registry, and the India's Clinical Trial Registry**

1821: "kidney transplantation"

Also searched for Kidney Transplants, Renal transplant, and Renal Transplantation in ClinicalTrials.gov

802: treg

Also searched for Regulatory T cell and T-regulatory cell in ClinicalTrials.gov

1638: sirolimus or rapamycin

Also searched for Everolimus, Sirolimus, RAD 001, Temsirolimus, mTOR inhibitor, cci779, Rapamune, Afinitor, Torisen, Certican, Zortess in ClinicalTrials.gov

1342: tacrolimus

Also searched for Prograf and FK 506 in ClinicalTrials.gov

1160: cyclosporine

Also searched for Restasis and Neoral in ClinicalTrials.gov

56: belatacept

Also searched for Nulojix and Bms-224818 in ClinicalTrials.gov

5638: creatinine

694: "acute rejection"

**DART Europe E-Theses**

215: "kidney transplantation"

176: "renal transplantation"

341: treg

80: sirolimus or rampamycin

76: everolimus

171: tacrolimus

156: cyclosporine

5: belatacept

428: creatinine

99: "acute rejection"

**Open Access Theses and Dissertations**

609: "kidney transplantation"

447: "renal transplantation"

1146: treg

1399: sirolimus or rapamycin

140: everolimus

410: tacrolimus

508: cyclosporine

14: belatacept

1788: creatinine

242: "acute rejection"

**Meeting abstracts archives**

**The American Association of Immunologists Annual Meeting 2006 to 2017, the European Congress of Immunology 2006, 2009, 2012 and 2015, the American Transplant Congress 2002-2005, 2007-2013, 2015-2017, the World Transplant Congress 2006 and 2014, the European Society for Organ Transplantation (ESOT) Congress 1997, 1999, 2001, 2005, 2007, 2009, 2011, 2013, 2015 and 2017, the Transplantation Society (TTS) International Congress 1998, 2000, 2002, 2004, 2006, 2008, 2010, 2012, 2014 and 2016**

12: manual searches.
